# lncRNA DLX6-AS1 Promotes Myocardial Ischemia-Reperfusion Injury through Mediating the miR-204-5p/FBXW7 Axis

**DOI:** 10.1155/2023/9380398

**Published:** 2023-01-10

**Authors:** Fanshun Wang, Yuan Wu

**Affiliations:** ^1^Department of Cardiac Surgery, Zhongshan Hospital of Fudan University, Shanghai 200032, China; ^2^Department of Cardiovascular Medicine, People's Hospital of Ningxia Hui Autonomous Region, The First Clinical College of Northwest University for Nationalities, Yinchuan, Ningxia Hui Autonomous Region 750002, China

## Abstract

Myocardial ischemia-reperfusion (IR) injury is the restoration of blood flow post ischemia, which threatens the human life. Long noncoding RNA distal-less homeobox 6 antisense 1 (DLX6-AS1) has been found to take part in the IR-induced cerebral injury. Here, we determined the functional role of DLX6-AS1 in IR-induced myocardial injury. We ligated the left anterior descending coronary artery of rats to induce IR injury. IR injury rats exhibited severe tissue damage and increase of infraction size. The levels of lactate dehydrogenase (LDH), creatine kinase (CK), proinflammatory factors including MCP-1, IL-6, and IL-1*β*, and cell apoptosis were also enhanced in IR rats, indicating that IR induced significant myocardial injury in rats. DLX6-AS1 expression was elevated in the myocardial tissues of IR injury rats, while DLX6-AS1 deficiency alleviated IR-induced myocardial injury in rats by reducing inflammatory response and cell apoptosis. Moreover, rat embryonic cardiomyocyte cell line H9c2 was subjected to hypoxia reoxygenation (HR). DLX6-AS1 was upregulated in the HR-treated H9c2 cells, and DLX6-AS1 enhanced the expression of F-box and WD40 repeat domain-containing 7 (FBXW7) by sponging miR-204-5p. Inhibition of DLX6-AS1 inhibited inflammatory response and cell apoptosis in H9c2 cells via miR-204-5p/FBXW7 axis. In conclusion, this work demonstrates that DLX6-AS1 accelerates myocardial IR injury through regulating miR-204-5p/FBXW7 axis. Thus, this work provides a novel ceRNA DLX6-AS1/miR-204-5p/FBXW7 axis in myocardial IR injury, and DLX6-AS1 may be a potential target for the treatment of myocardial IR injury.

## 1. Introduction

Acute myocardial infarction (AMI) is a common and frequently occurring disease in the cardiovascular system [1]. Timely reperfusion therapy is the most effective treatment for AMI patients, which can save more cardiomyocytes that are on the verge of necrosis [2, 3]. However, a lot of cardiomyocytes died owing to reperfusion injury after revascularization. Although AMI patients survived form myocardial ischemic necrosis, they still have severe cardiac dysfunction, which affects the long-term prognosis of AMI patients [4]. This kind of reperfusion therapy after ischemia restores the blood supply but aggravates the tissues injury, which is called ischemia-reperfusion (IR) injury [5]. The main feature of IR is the severe mucosal and submucosal osmotic inflammation, which leads to the necrosis of endothelial cells [6].

Long noncoding RNA (lncRNA) has been proposed as a key regulator of various biological processes [7]. Recent evidences indicate that lncRNA regulates various physiological processes such as cell differentiation, proliferation, apoptosis, and inflammation [8–10]. lncRNA distal-less homeobox 6 antisense 1 (DLX6-AS1) plays a carcinogenic role in lung adenocarcinoma, ovarian cancer, and other cancers [11, 12]. Moreover, DLX6-AS1 is upregulated in cerebral IR injury, and inhibition of DLX6-AS1 significantly reduces apoptosis in neuronal cells [13]. However, little is known about DLX6-AS1 in myocardial IR injury.

lncRNA and microRNA (miRNA) are widely involved in competitive endogenous RNA (ceRNA) model. lncRNA relieves the inhibitory effect of miRNA on the downstream target mRNA by sponging miRNA [14]. Many miRNAs have become important regulators in the physiological and pathological processes of cardiovascular diseases and participate in regulating cardiac functions, such as contraction, cardiac growth, and morphogenesis [15, 16]. Previous study has revealed that the expression of miR-204-5p is lower in the myocardial tissue of the myocardial IR injury mouse model. KCNQ1OT1 binds to miR-204-5p and aggravates myocardial IR injury in mice by upregulating LGALS3, which provides a new idea for the treatment of myocardial IR injury [17]. Whether miR-204-5p has a functional role in IR-induced myocardial injury remains unclear. Additionally, Bioinformatical analysis (Starbase) revealed that miR-204-5p may be a downstream target of DLX6-AS1, and there may be binding sites between miR-204-5p and oxidized low-density lipoprotein receptor-1 (FBXW7). A previous study has uncovered that FBXW7 expression was elevated in myocardial IR injury. Inhibition of FBXW7 significantly alleviated inflammatory response, cell apoptosis, and IR-induced myocardial injury in mice [18, 19]. Thus, we speculated that DLX6-AS1 may alleviate myocardial IR injury by regulating miR-204-5p/FBXW7 axis.

In this work, we constructed a rat model of IR injury and determined the impact of DLX6-AS1 inhibition on IR injury in rats. Next, we further verified the mechanism of action of DLX6-AS1/miR-204-5p/FBXW7 axis in myocardial IR injury *in vitro*.

## 2. Materials and Methods

### 2.1. IR Injury Rat Model

Wistar male rats with 14 weeks were purchased from the Shanghai SLAC Laboratory Animal Co., Ltd (China). All protocols were carried out under the authorization of the Ethics Committee of Zhongshan Hospital of Fudan University.

IR injury rat model was established by ligation of the left anterior descending (LAD) coronary artery as previously described [20]. Briefly, rats were anesthetized with intraperitoneal injection of 45 mg/kg pentobarbitone. Subsequently, the trachea of rats was carefully exposed. Then, a rodent ventilator TOPO Dual Mode Ventilator (Kent Scientific Corporation, Torrington, CT, USA) was connected to the trachea for establishment of mechanical ventilation. The left ventricle was exposed from the chest under the rats with steady breathing. The LAD coronary artery was ligated with a 6-0 suture to induce ischemia for 30 min. Subsequently, the ligature was cut to mimic reperfusion. After that, the chest was sutured utilizing 6-0 Prolene sutures. In sham-operated rats, the procedure was the same except for artery ligation. The rats were sacrificed 6 h after the LAD ligation-reperfusion intervention. After the rats were sacrificed, tissues about 5 mm around the infarct area were selected for subsequent experiments.

For induce knockdown of DLX6-AS1, rats were injected with 1 × 10^12^ vg/mL of AAV9-sh-DLX6-AS1 at multiple sites of myocardium (basal anterior, midanterior, midlateral, anterior apical, and lateral apical) using a microsyringe. Rats were injected with AAV9-sh-Scramble as control. The LAD coronary artery of rats was ligated after 24 hours post the injection. The AAV9-adenovirus particles containing short hairpin RNA (shRNA) specifically targeting DLX6-AS1 (AAV9-sh-DLX6-AS1) and scrambled RNA (AAV9-sh-Scramble) were obtained from GenePharma (Shanghai, China).

### 2.2. Histological Analysis

Paraffin sections (5 *μ*m) of myocardial tissues were subjected to dewaxing and hydration. Then, the sections were stained with hematoxylin and eosin sequentially using Hematoxylin-Eosin (HE) Staining Kit (Beyotime, Shanghai, China). For detection of apoptotic cells in myocardial tissues, the paraffin sections were stained with TUNEL Apoptosis Assay Kit (Beyotime, Shanghai, China). The nucleus was stained with DAPI (Beyotime, Shanghai, China). Finally, the pathological changes and apoptotic cells in myocardial tissues were observed under optical or fluorescence microscope.

### 2.3. 2,3,5-Triphenyte-Trazoliumchloride (TTC) Staining

The infarction size of myocardial tissues was determined as previously reported [20]. The hearts were separated form rats, and the left ventricle was sliced into sections with 2 mm perpendicular to the long axis of heart. The sections were incubated with 1% TTC (Solarbio, Beijing, China) at 37°C for 60 min. The sections were sealed in closed freezer bag containing PBS to avoid oxidation of samples. The red part with TTC staining was ischemic but viable tissue, and the unstained area indicated the infarction area of myocardial tissues. The infarction size of myocardial tissues was analyzed using Image Pro Plus 6.0.

### 2.4. Enzyme-Linked Immunosorbent Assay (ELISA)

The levels of lactate dehydrogenase (LDH), creatine kinase (CK), monocyte chemoattractant protein-1 (MCP-1), IL-6, and IL-1*β* in the serum of rats were assessed utilizing Rat ELISA Kit (Jianglaibio, Shanghai, China) following the protocol of kit. The absorbance was examined on a microplate reader (Tecan, San Jose, CA, USA).

### 2.5. Cell Culture

The rat embryonic cardiomyocyte cell line H9c2 (CCTCC, Wuhan, China) was cultured in Dulbecco's Modified Eagle Medium (DMEM; Gibco BRL, Grand Island, NY, USA) containing 10% fetal bovine serum (FBS; Gibco) and 100 *μ*g/mL penicillin/streptomycin at 37°C and 5% CO_2_. For inducing IR through hypoxia reoxygenation (HR) treatment, H9c2 cells were cultured in serum- and glucose-free DMEM in a hypoxic chamber for 6 h, followed by incubation in DMEM containing 10% FBS in normal oxygen environments.

### 2.6. Cell Transfection

The pcDNA3.1 carrying DLX6-AS1 (pc-DLX6-AS1) and small interfering RNA specially targeting DLX6-AS1 (si-DLX6-AS1) were used for overexpressing or knocking down DLX6-AS1. The oligonucleotides miR-204-5p mimic and miR-204-5p inhibitor were used to upregulate and downregulate miR-204-5p. The plasmids pc-DNA, NC-mimic, and inh-NC were served as control. All the plasmids were obtained from the GenePharma. H9c2 cells were transfected with the plasmids utilizing Lipofectamine 2000 Transfection Reagent (Invitrogen, Carlsbad, CA, USA).

### 2.7. Quantitative Real-Time PCR (qRT-PCR)

Myocardial tissues and H9c2 cells were treated with TRIzol reagent (Invitrogen) to extract total RNA. PrimeScript RT Master Mix (TaKaRa, Tokyo Japan) was utilized to synthesize complementary DNA from total RNA. The relative expression of mRNAs was assessed by using TB Green Premix Ex Taq II (TaKaRa). The primer sequence (5′-3′) was shown as follows: DLX6-AS1-forward: CCA CCC ACT GAG AGA AGA GG, DLX6-AS1-reverse: CCT CCA AGC AAT TGT CCA GT; FBXW7-forward: GTT CCG CTG CCT AAT CTT CCT, FBXW7-reverse: CCC TTC AGG GAT TCT GTG CC; miR-204-5p-forward: TCC GTA TCC TAC TGT T, miR-204-5p-reverse: GCA GGG TCC GAG GTA TTC; MCP-1-forward: TCT GGG CCT GTT GTT CAC AGT, MCP-1-reverse: TGC TGC TGG TGA TTC TCT TGT AGT; IL-6-forward: CTC TCC GCA AGA GAC TTC CAG, IL-6-reverse: TTC TGA CAG TGC ATC ATC GCT; IL-1*β*-forward: ACA GAT GAA GTG CTC CT; IL-1*β*-reverse: AAG ATA GGT TCT TCT TCA; GAPDH-forward: GTC AGC CGC ATC TTC TTT TG, GAPDH-reverse: GCG CCC AAT ACG ACC AAA TC; U6-forward: CTC GCT TCG GCA GCA CA, U6-reverse: AAC GCT TCA CGA ATT TGC GT. The relative expression of lncRNA and mRNA was normalized to GAPDH, and miRNA expression was normalized to U6. The results were analyzed using 2^-∆∆CT^ method for quantification.

### 2.8. Western Blot (WB)

Total Protein Extraction Kit (Solarbio, Beijing, China) and BCA Protein Assay Kit (Solarbio, Beijing, China) were utilized to extract proteins from myocardial tissues or H9c2 cells and measure protein concentration. Proteins from each sample were separated by SDS-PAGE gel electrophoresis and then transferred onto the PVDF membranes. The membranes were stained with the primary antibody, Bax (Cat: ab32503; 1 : 1000 dilution), Bcl-2 (Cat: ab194583; 1 : 1000 dilution), FBXW7 (Cat: ab109617; 1 *μ*g/mL dilution), or GAPDH (Cat: ab181602; 1 : 1000 dilution) 4°C for 12 h and then incubated with goat antirabbit HRP-IgG (Cat: ab6721; 1 : 2000 dilution) at 37°C for 1 h. These antibodies were obtained from Abcam (Cambridge, MA, USA). The data was analyzed by Image J software. The expression of protein was normalized to the internal reference gene GAPDH.

### 2.9. Luciferase Reporter Assay

Starbase online tool uncovered that there may be binding sites between DLX6-AS1 and miR-204-5p or betweenmiR-204-5p and FBXW7. To verify this speculation, the luciferase reporter vectors pmir-GLO containing wild type (WT) or mutant type (Mut) of DLX6-AS1 and FBXW7 were constructed by GenePharma. H9c2 cells were transfected with WT/Mut of luciferase reporter vectors and miR-204-5p mimic or NC-mimic using Lipofectamine 2000 Transfection Reagent. Finally, Luciferase Assay System (Ambion, Austin, TX, USA) was used to assess the relative luciferase activity of H9c2 cells.

### 2.10. RNA-Binding Protein Immunoprecipitation (RIP)

RIP assay was carried out to determine whether DLX6-AS1 and miR-204-5p interacted with Ago-2 to form a RNA-induced silencing complex (RISC) according to reported work [21]. H9c2 cells transfected with pc-DLX6-AS1 were incubated with polysome lysis buffer on ice for 5 min. The cell lysates were incubated with 10 *μ*g anti-pan-Ago IgG 2A8 (Cat: MABE56; Merck Millipore, Billerica, MA, USA) and conjugated with Dynabeads Protein G (Thermo Fisher Scientific, Waltham, MA, USA) at overnight rotating at 4°C. IgG1 isotype control (Cat: 21275511; ImmunoTools, Friesoythe, Germany) coated with Dynabeads Protein G was used as control. Subsequently, the cell lysates were incubated with 50 *μ*L glycine (pH 2.3) at room temperature for 15 min to elute protein-RNA complexes from the beads. The protein-RNA complexes were lysed with proteinase K. Finally, miRNeasy Mini Kit (Qiagen, Düesseldorf, Germany) was used to purify RNA following the manufacturer's recommendations. The purified RNA was served as template to detect DLX6-AS1 and miR-204-5p expression by performing qRT-PCR. Cell lysates were served as Input group.

### 2.11. Flow Cytometry

Flow cytometry was done to measure apoptotic cells in H9c2 cells utilizing Annexin V-FITC Apoptosis Detection Kit (Beyotime, Shanghai, China) on a BD FACSCalibur (BD Bioscience, San Jose, USA). H9c2 cells were cultured for 24 h. After washing with PBS for 3 times, cells were stained with Annexin V-FITC and PI in the dark. Finally, the apoptotic cells were detected by flow cytometry analysis.

### 2.12. Statistical Analysis

Each assay was performed for 3 times. The representative data were presented and expressed as mean ± standard deviation. Statistical analysis was carried out utilizing SPSS 22.0 statistical software (IBM, Armonk, NY, USA). Two-tailed Student's *t*-test (between two groups) and one-way ANOVA (among multiple groups) were utilized to analyze the statistical differences. *P* < 0.05 indicates significant differences.

## 3. Results

### 3.1. The Expression of DLX6-AS1 Was Decreased in IR Injury Rats

**Figure 1:**
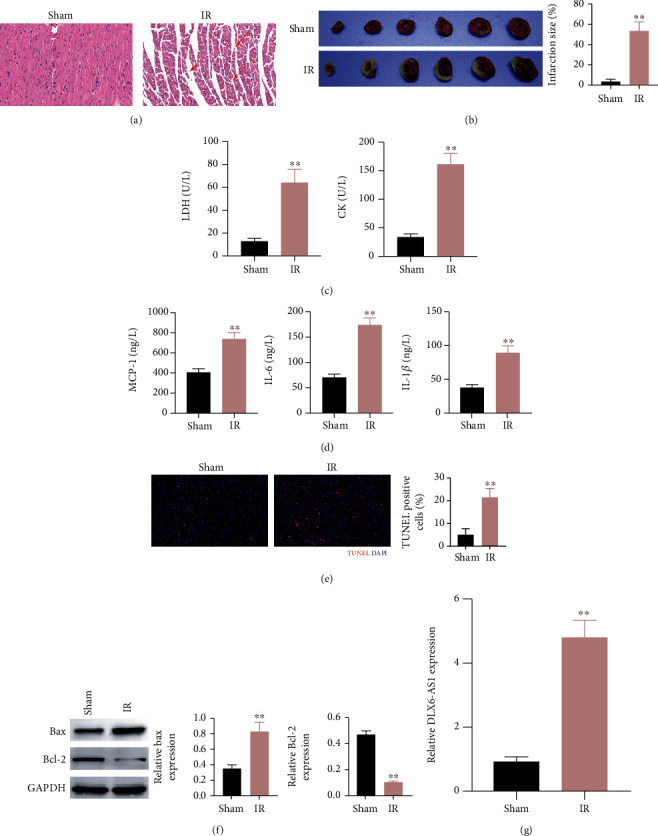
DLX6-AS1 expression was increased in the myocardial tissues of IR injury rats. IR rats were established by LAD ligation. Sham-operated rats were served as control. (a) The pathological changes of myocardial tissues were analyzed by HE staining. (b) Infarction size of myocardial tissues was examined by TTC staining. (c) The levels of LDH and CK in the serum were detected by ELISA. (d) The levels of MCP-1, IL-6, and IL-1*β* in the serum were detected by ELISA. (e) Apoptosis in myocardial tissues was detected by TUNEL staining. (f) The expression of Bax and Bcl-2 in myocardial tissues was analyzed by WB. (g) The DLX6-AS1 mRNA expression was examined through qRT-PCR. ^∗∗^*P* vs. Sham.

In order to determine the function of DLX6-AS1 in IR injury, a rat model of IR injury was constructed by LAD ligation. HE and TTC staining revealed that IR rats exhibited a severe damage and an increase of infarction size in the myocardial tissues compared with sham-operated rats (Figures 1(a) and 1(b)). Moreover, we detected the level of myocardial enzyme spectrum in the serum by ELISA, showing that LDH and CK were increased in IR rats (Figure 1(c)). The proinflammatory factors such as MCP-1, IL-6, and IL-1*β* were also enhanced in the serum of IR rats as compared with sham-operated rats (Figure 1(d)). Furthermore, cell apoptosis in the myocardial tissues of rats was measured by TUNEL staining, revealing that the apoptotic cells were increased in IR rats (Figure 1(e)). Compared with that in sham-operated rats, Bax protein was upregulated, while Bcl-2 protein was downregulated in myocardial tissues of IR rats (Figure 1(f)). Interestingly, DLX6-AS1 expression was upregulated in the myocardial tissues of IR rats (Figure 1(g)). Thus, these data indicated that the upregulation of DLX6-AS1 may be associated with myocardial IR injury.

### 3.2. Inhibition of DLX6-AS1 Attenuated IR-Induced Myocardial Injury

**Figure 2:**
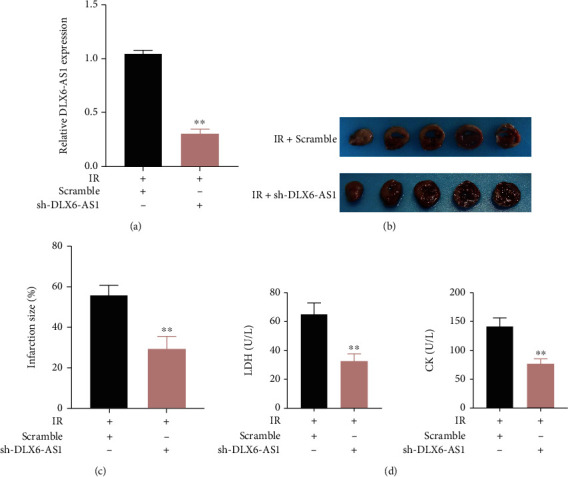
DLX6-AS1 downregulation alleviated myocardial infarction and myocardial damage in IR rats. IR rats were established by LAD ligation, which were then transfected with sh-DLX6-AS1 or Scramble. (a) The expression of DLX6-AS1 in the myocardial tissues was examined by qRT-PCR. (b and c) Infarction size of myocardial tissues was examined by TTC staining. (d) The levels of LDH and CK in the serum were assessed by ELISA. ^∗∗^*P* vs. IR + Scramble.

Next, we investigated whether inhibition of DLX6-AS1 can attenuate IR injury in rats. DLX6-AS1 was silenced in IR rats, and the results showed that DLX6-AS1 was severely downregulated in the myocardial tissues of IR rats following injection of sh-DLX6-AS1 (Figure 2(a)). Then, the myocardial infarction of IR rats was examined by TTC staining. As shown in Figures 2(b) and 2(c), the infarction size in myocardial tissues of IR rats was severely decreased. We also found that silencing DLX6-AS1 caused a decrease in the levels of LDH and CK in the serum of IR rats (Figure 2(d)).

**Figure 3:**
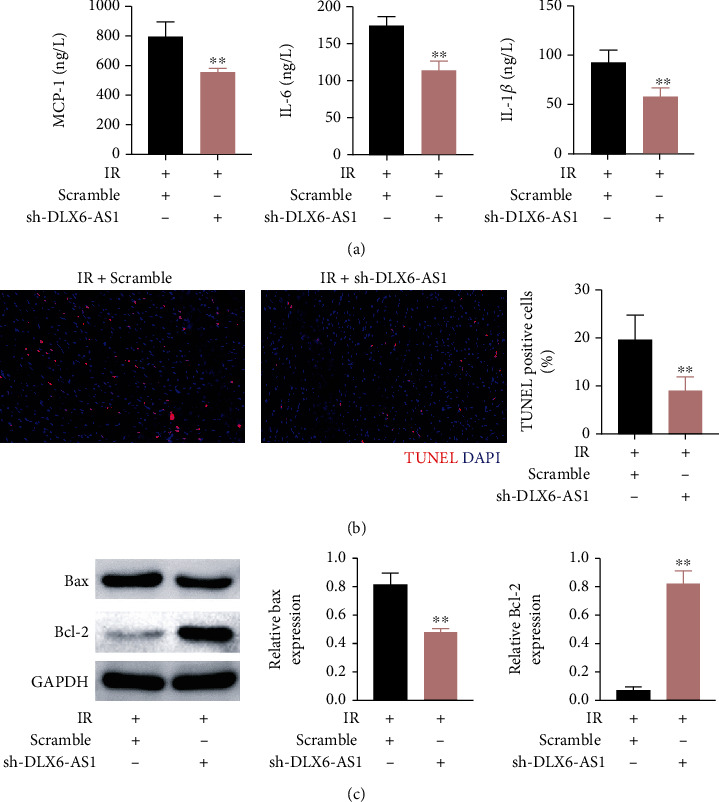
DLX6-AS1 downregulation repressed inflammatory response and cell apoptosis in IR rats. IR rats were established by LAD ligation, which were then transfected with sh-DLX6-AS1 or Scramble. (a) The levels of MCP-1, IL-6, and IL-1*β* in the serum were detected by ELISA. (b) Apoptosis in myocardial tissues was detected by TUNEL staining. (c) The expression of Bax and Bcl-2 in myocardial tissues was analyzed by WB. ^∗∗^*P* vs. IR + Scramble.

Additionally, ELISA results revealed that the inflammatory cytokines including MCP-1, IL-6, and IL-1*β* in the serum of IR rats were decreased by DLX6-AS1 deficiency (Figure 3(a)). TUNEL staining and WB assays were carried out to assess apoptotic cells and the expression of apoptosis-related proteins in myocardial tissues of IR rats. Silence of DLX6-AS1 reduced the number of apoptotic cells through downregulating Bax and upregulating Bcl-2 in IR rats (Figures 3(b) and 3(c)).

Taken together, DLX6-AS1 deficiency reduced myocardial infarction, inflammatory response, and cell apoptosis in IR rats.

### 3.3. DLX6-AS1 Promoted FBXW7 Expression by Competitively Binding to miR-204-5p

**Figure 4:**
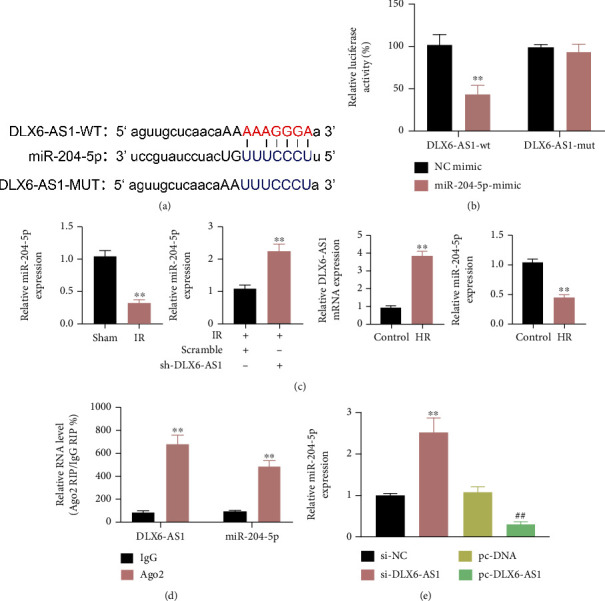
DLX6-AS1 interacted with miR-204-5p and repressed miR-204-5p expression. (a) The predicted binding sites between DLX6-AS1 and miR-204-5p were presented. (b) The interaction between DLX6-AS1 and miR-204-5p in H9c2 cells was verified by luciferase reporter assay. (c) IR rats were established by LAD. Sham-operated rats were served as control. H9c2 cells were subjected to HR. Normal H9c2 cells were served as control. The expression of miR-204-5p and DLX6-AS1 in the myocardial tissues and H9c2 cells was examined by qRT-PCR. (d) RIP assay was carried out utilizing an anti-Ago2 antibody in H9c2 cells transfected with pc-DLX6-AS1 to detect the expression of DLX6-AS1 and miR-204-5p through qRT-PCR. (e) The qRT-PCR was carried out to assess the expression of miR-204-5p in H9c2 cells transfected with si-NC, si-DLX6-AS1, pc-DNA, or pc-DLX6-AS1.^∗∗^*P* vs. NC-mimic, Sham, Control, IgG, si-NC; ^##^*P* vs. pc-DNA.

Bioinformatics software Starbase revealed that there may be binding sites between DLX6-AS1 and miR-204-5p (Figure 4(a)). Luciferase reporter assay confirmed that DLX6-AS1 interacted with miR-204-5p in H9c2 cells (Figure 4(b)). Furthermore, the expression of miR-204-5p and DLX6-AS1 in IR rats and HR-treated H9c2 cells was examined by qRT-PCR. Compared with sham-operated rats, miR-204-5p expression was decreased in IR rats and HR-treated H9c2 cells. DLX6-AS1 was upregulated in HR-treated H9c2 cells. Furthermore, miR-204-5p expression was increased after silencing DLX6-AS1 (Figure 4(c)). In addition, both DLX6-AS1 and miR-204-5p interacted with Ago-2 to form a RISC (Figure 4(d)). The mRNA expression of miR-204-5p was significantly enhanced in H9c2 cells transfected with si-DLX6-AS1, while miR-204-5p expression was severely decreased in H9c2 cells transfected with pc-DLX6-AS1 (Figure 4(e)).

**Figure 5:**
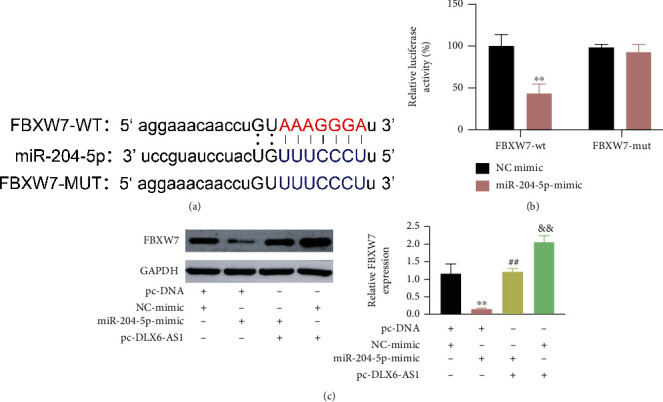
DLX6-AS1 promoted FBXW7 expression by sponging miR-204-5p. (a) The predicted binding sites between miR-204-5p and FBXW7 were presented. (b) The interaction between miR-204-5p and FBXW7 in H9c2 cells was verified by luciferase reporter assay. (c) H9c2 cells were transfected with pc-DLX6-AS1 or pc-DNA and miR-204-5p-mimic or NC-mimic. FBXW7 expression in the H9c2 cells was examined by WB. ^∗∗^*P* vs. NC-mimic, pc-DNA + NC-mimic; ^##^*P* vs. pc-DNA + miR-204-5p-mimic; ^&&^*P* vs. miR-204-5p-mimic + pc-DLX6-AS1.

Subsequently, bioinformatics analysis uncovered that FBXW7 may be a downstream target of miR-204-5p, which was verified by luciferase reporter assay (Figures 5(a) and 5(b)). Moreover, FBXW7 expression was repressed by miR-204-5p overexpression and increased by DLX6-AS1 upregulation in H9c2 cells. DLX6-AS1 upregulation reversed miR-204-5p mimic-mediated inhibitory effect on FBXW7 expression (Figure 5(c)).

In a word, DLX6-AS1 functioned as a ceRNA to interact with miR-204-5p, which contributed to regulate the expression of its down-stream target FBXW7.

### 3.4. DLX6-AS1 Deficiency Alleviated IR-Induced Myocardial Injury by Regulating miR-204-5p

**Figure 6:**
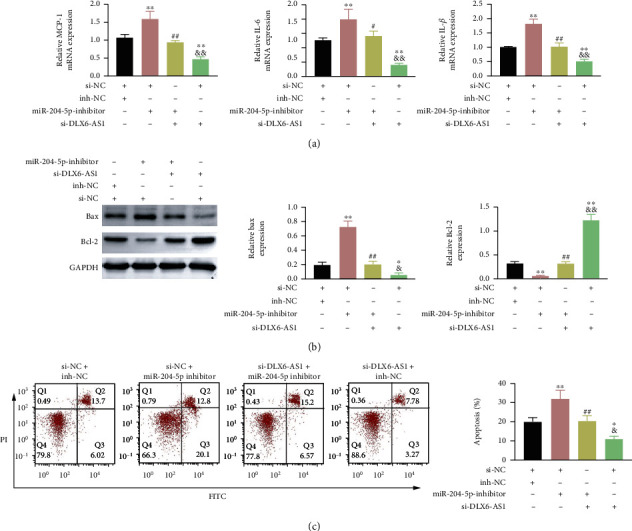
DLX6-AS1 deficiency repressed inflammatory response and cell apoptosis in HR-treated H9c2 cells by regulating miR-204-5p. H9c2 cells were transfected with si-DLX6-AS1 or si-NC and miR-204-5p inhibitor or inh-NC, followed by HR treatment. (a) The expression of MCP-1, IL-6, and IL-1*β* in the H9c2 cells was detected by qRT-PCR. (b) The expression of Bax and Bcl-2 in the H9c2 cells was assessed by WB. (c) Apoptosis of H9c2 cells was measured through flow cytometry. ^∗∗^*P* vs. si-NC + inh-NC; ^##^*P* vs. si-NC + miR-204-5p inhibitor; ^&&^*P* vs. miR-204-5p inhibitor + si-DLX6-AS1.

Finally, the functional role of DLX6-AS1 was verified *in vitro*. Both DLX6-AS1 and miR-204-5p were silenced in H9c2 cells, which were then treated with HR. Inhibition of miR-204-5p significantly enhanced MCP-1, IL-6, and IL-1*β* expression in HR-treated H9c2 cells. In contrast, DLX6-AS1 silencing led a decrease of these inflammatory factors in HR-treated H9c2 cells. Inhibition of miR-204-5p reversed si-DLX6-AS1-mediated anti-inflammatory effect in HR-treated H9c2 cells (Figure 6(a)). Moreover, the influence of knocking down DLX6-AS1 on apoptosis was investigated in HR-treated H9c2 cells by WB and flow cytometry. Knockdown of miR-204-5p enhanced Bax expression and repressed Bcl-2 expression in HR-treated H9c2 cells. Upregulation of DLX6-AS1 exerted an opposite effect on these apoptosis-related proteins, which was rescued by silencing DLX6-AS1 (Figure 6(b)). Apoptosis of HR-treated H9c2 cells was accelerated by miR-204-5p deficiency and decreased by DLX6-AS1 inhibition. However, knockdown of DLX6-AS1-mediated inhibition of apoptosis was abrogated by miR-204-5p inhibitor (Figure 6(c)). Thus, inhibition of DLX6-AS1 repressed inflammatory response and cell apoptosis in HR-treated H9c2 cells by regulating miR-204-5p.

It is also important that we discovered that the infraction size was reduced after silencing DLX6-AS1, but this effect could be reversed by overexpressing FBXW7 (Figure Supplementary A). Additionally, the decreased LDH and CK levels of mediated DLX6-AS1 knockdown were rescued by FBXW7 overexpression (Figure Supplementary B).

## 4. Discussion

Myocardial IR injury is a complex process, and many factors are involved in the damage of myocardial tissues [22]. In the past few decades, scholars attempt to clarify the molecular mechanism of myocardial IR injury and find the effective treatment to reduce the scope and extent of myocardial injury caused by IR [22].

DLX6-AS1 participates in various diseases. For instance, Liu et al. have revealed that DLX6-AS1 expression was elevated in the placenta tissues of preeclampsia [23]. DLX6-AS1 functions as ceRNA to regulate miR-149-5p/ERP44 axis, which contributes to aggravate preeclampsia progression. DLX6-AS1 is rich in the serum exosomes of cervical cancer patients, whose upregulation is positively related to the poor prognosis of cervical cancer [24]. DLX6-AS1 inhibits ANXA10 expression through ubiquitination-mediated degradation to accelerate hepatocellular carcinoma development, which can be attributed to the regulation on miR-513c/Cul4A axis [25]. Hu et al. have confirmed that DLX6-AS1 promoted I/R-induced cerebral neuron impairments by regulating miR-149-3p/BOK axis [13]. In the present work, the IR injury rats displayed a severe tissue damage and significant increase of the myocardial infarction size and cell apoptosis. The levels of serum myocardial enzyme spectrum LDH and CK and proinflammatory factors MCP-1, IL-6, and IL-1*β* were significantly increased in the myocardial tissues of IR injury rats. These data showed that IR injury rats had a significant myocardial damage, which was rescued by inhibition of DLX6-AS1 expression. Thus, DLX6-AS1 played a crucial role in the development of myocardial IR injury.

The miR-204-5p expression is lower in the myocardial tissue, and KCNQ1OT1/miR-204-5p/LGALS3 axis was involved in the myocardial IR injury in mice [17]. In this work, it was verified that DLX6-AS1 repressed miR-204-5p expression by targeting miR-204-5p. The expression of miR-204-5p was decreased in IR rats and HR-treated H9c2 cells. Both DLX6-AS1 and miR-204-5p interacted with Ago-2 to form a RISC. The assembly of RISC is the central link of RNAi and miRNA pathways, which has the activity of silencing the target mRNA [26]. Ago-2 is the core protein in RISC and has the ability to shear mRNA [27]. Moreover, miR-204-5p interacted with FBXW7, and inhibited FBXW7 expression. DLX6-AS1 rescued the miR-204-5p-mediated inhibition on FBXW7 expression. Thus, DLX6-AS1 elevated FBXW7 expression by inhibiting miR-204-5p expression, thereby can act as a ceRNA.

FBXW7 is a F-box WD40 protein and serves as a substrate recognition subunit of the SCF (SKP1/CUL1/F-box protein) E3 ubiquitin ligase complex. In recent years, a lot of work has found that miR-223-3p targets FBXW7 to regulate myocardial inflammation and apoptosis after myocardial infarction [18]. Additionally, miR322 modulates the FBXW7/notch pathway to affect cardioprotection against IR injury [19]. FBXW7 regulates EZH2-SIX1 signaling to accelerate pathological cardiac hypertrophy [28]. Moreover, miR-211-5p/FBXW7 axis relieves the myocardial ischemia injury stimulated by IR treatment [29]. Besides, miR-195-5p affects MFN2 and FBXW7 to facilitate cardiomyocyte hypertrophy [30]. Consistently, the role of FBXW7 in IR injury was also confirmed in this study. DLX6-AS1 deficiency repressed inflammatory response and cell apoptosis in HR-treated H9c2 cells through regulating miR-204-5p/FBXW7 axis.

## 5. Conclusion

This work demonstrated that lncRNA DLX6-AS1 promoted FBXW7 expression by sponging miR-204-5p, which contributed to accelerating myocardial IR injury. Thus, this work provides a novel ceRNA DLX6-AS1/miR-204-5p/FBXW7 axis in myocardial IR injury, and DLX6-AS1 may be a potential target for myocardial IR injury treatment.

## Data Availability

All data generated or analyzed during this study are included in this published article.

## Supplementary Materials

Supplementary Materials Figure Supplementary. (A) Infarction size of myocardial tissues was examined by TTC staining. (B) The levels of LDH and CK in the serum were detected by ELISA. ^∗∗^*P* vs. IR + AAV-scramble+empty; ^##^*P* vs. IR + AAV-sh-DLX6-AS1 + empty.
