# Supplementary figures and images for: lncRNA DLX6-AS1 Promotes Myocardial Ischemia-Reperfusion Injury through Mediating the miR-204-5p/FBXW7 Axis
Source: Mediators Inflamm. 2023 Jan 10;2023:9380398. doi: 10.1155/2023/9380398 (PMC9845044; doi:10.1155/2023/9380398)

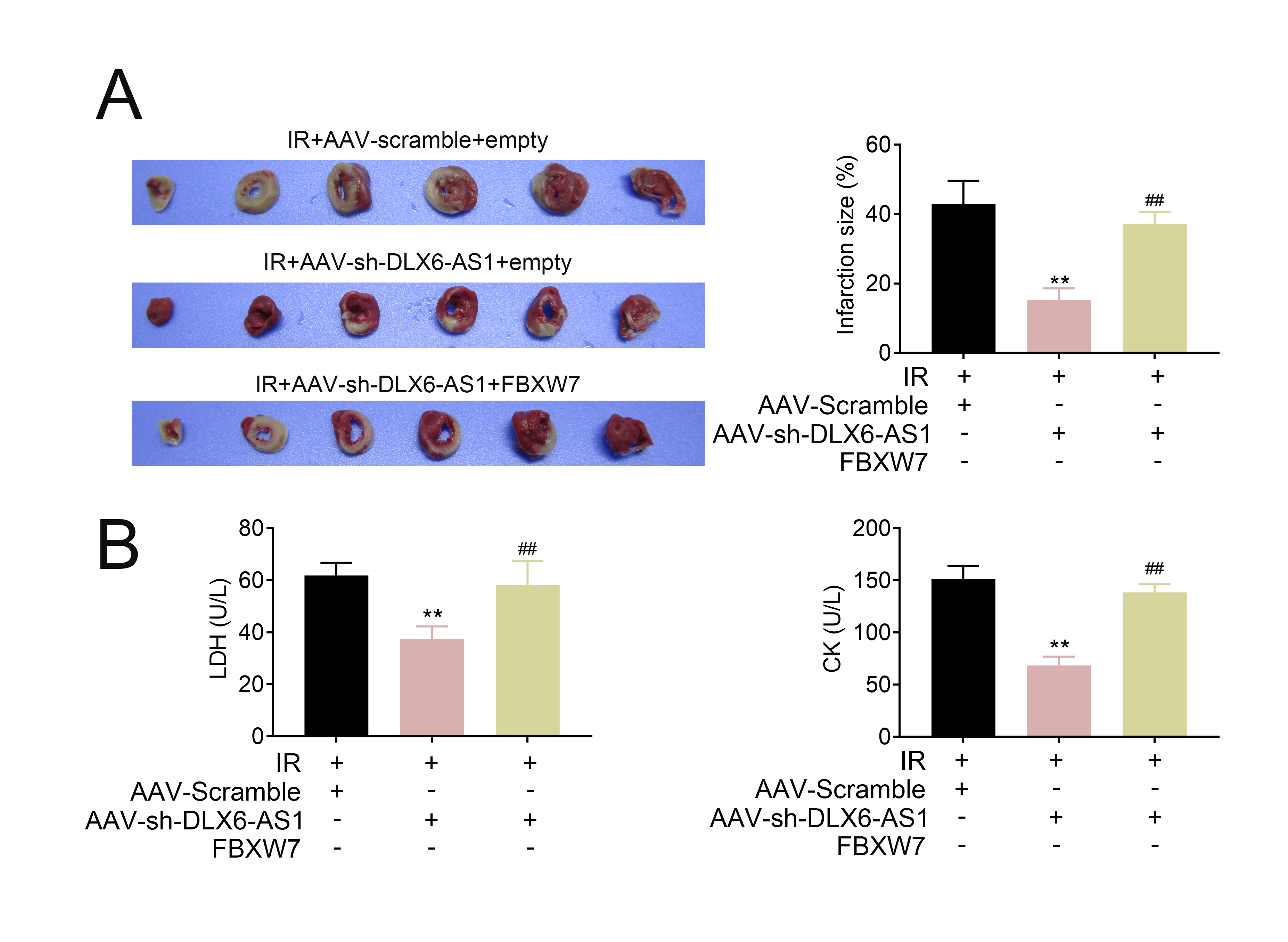

Supplement: Supplementary Materials — Figure Supplementary. (A) Infarction size of myocardial tissues was examined by TTC staining. (B) The levels of LDH and CK in the serum were detected by ELISA. ∗∗P vs. IR + AAV-scramble+empty; ##P vs. IR + AAV-sh-DLX6-AS1 + empty. [file 9380398.f1.jpg]
